# Network Properties of Local Fungal Communities Reveal the Anthropogenic Disturbance Consequences of Farming Practices in Vineyard Soils

**DOI:** 10.1128/mSystems.00344-21

**Published:** 2021-05-04

**Authors:** Rüdiger Ortiz-Álvarez, Héctor Ortega-Arranz, Vicente J. Ontiveros, Miguel de Celis, Charles Ravarani, Alberto Acedo, Ignacio Belda

**Affiliations:** a Biome Makers Inc., West Sacramento, California, USA; b Theoretical and Computational Ecology, Centre for Advanced Studies of Blanes (CEAB), Spanish Research Council (CSIC), Blanes, Spain; c Department of Genetics, Physiology and Microbiology, Complutense University of Madrid, Madrid, Spain; California State University, Northridge

**Keywords:** agroecosystems, emergent properties, fungal communities, local networks

## Abstract

Agroecosystems are human-managed ecosystems subject to generalized ecological rules. Understanding the ecology behind the assembly and dynamics of soil fungal communities is a fruitful way to improve management practices and plant productivity. Thus, monitoring soil health would benefit from the use of metrics that arise from ecological explanations that can also be informative for agricultural management. Beyond traditional biodiversity descriptors, community-level properties have the potential of informing about particular ecological situations. Here we assess the impact of different farming practices in a survey of 350 vineyard soils from the United States and Spain by estimating network properties based on spatial associations. Our observations using traditional approaches show results concurring with previous literature: the influence of geographic and climatic factors on sample distributions, or different operational taxonomic unit (OTU) compositions depending on agricultural managements. Furthermore, using network properties, we observe that fungal communities ranged from dense arrangements of associations to a sparser structure of associations, indicating differential levels of niche specialization. We detect fungal arrangements capable of thriving in wider or smaller ranges of temperature, revealing that niche specialization may be a critical soil process impacting soil health. Low-intervention practices (organic and biodynamic managements) promoted densely clustered networks, describing an equilibrium state based on mixed collaborative communities. In contrast, conventionally managed vineyards had highly modular sparser communities, supported by a higher coexclusion proportion. Thus, we hypothesize that network properties at the community level may help to understand how the environment and land use can affect community structure and ecological processes in agroecosystems.

**IMPORTANCE** Soil fungal communities play a key role in agroecosystem sustainability. The complexity of fungal communities, at both taxonomic and functional levels, makes it difficult to find clear patterns connecting community composition with ecosystem function and to understand the impact of biotic (interspecies interactions) and abiotic (e.g., climate or anthropogenic disturbances) factors on it. Here we combine network analysis methods and properties, proposing a novel analytical approach: to infer ecological properties from local networks, which we apply to the study of fungal communities in vineyard soils. We conclude that different levels of farming intensification may lead to different ecological strategies in soil fungal communities settled by particular association arrangements.

**Author Video**: An author video summary of this article is available.

## INTRODUCTION

Fungal biodiversity is a major component of soil ecosystems. The community composition, characteristics, and behavior have consequences for the whole ecosystem where these organisms thrive. In summary, fungi are directly involved in ecosystem services (organic matter transformations, nutrient cycling, biocontrol agents) and have effects on plant and crop physiology through fungus-plant interactions (i.e., they generate bioactive phytochemicals, and they regulate pathogen occurrence) (1). As an attempt to understand the underlying ecological processes explaining how microbial communities are shaped, most studies currently focus on correlative evidence between specific taxon abundance, diversity metrics, and environmental factors or community phenotypes (2). And although valuable, it has been argued that this strategy does not allow understanding the underlying ecological mechanisms by which communities react to environmental factors or by which these communities organize to perform an ecosystem-level process (3). Hence, developing a strategy to mechanistically understand the fungal component of the soil microbiome has general implications in monitoring soil health and may be of particular interest for guiding management strategies of agroecosystems. In particular, developing and understanding measurable metrics can be a critical step for future microbiome monitoring applications, such as in sustainable farming or food production (4). Indeed, smart farming demands new biomarkers to monitor soil health (see U.S. Department of Agriculture [USDA] definition at https://www.nrcs.usda.gov/wps/portal/nrcs/main/soils/health/), since comprehensive information is often inaccessible to land managers (5), and a single universal methodology to measure soil quality and health based on the microbiome does not exist yet (6, 7) despite notable efforts (8, 9).

In this sense, we aim at suggesting an approach that can be both theoretically relevant and also informative for land managers. Ecological communities are often defined by functional traits, which result from the aggregation of taxon characteristics (10, 11) or through properties arising from specific combinations of taxa (12, 13). These community-level properties are characteristics that are proxies of ecological processes, which further determine species pools, trophic fluxes (3), or ecosystem maturity (11). Exploring them may lead to predictions of how communities would behave under concrete circumstances. Methodologically, there have been different strategies to measure such properties in microbiomes, but here we aimed at describing community-level properties by combining large-scale associational networks (from now on, metawebs) and a methodological innovation to split inferred associations into local communities.

**FIG 1:**
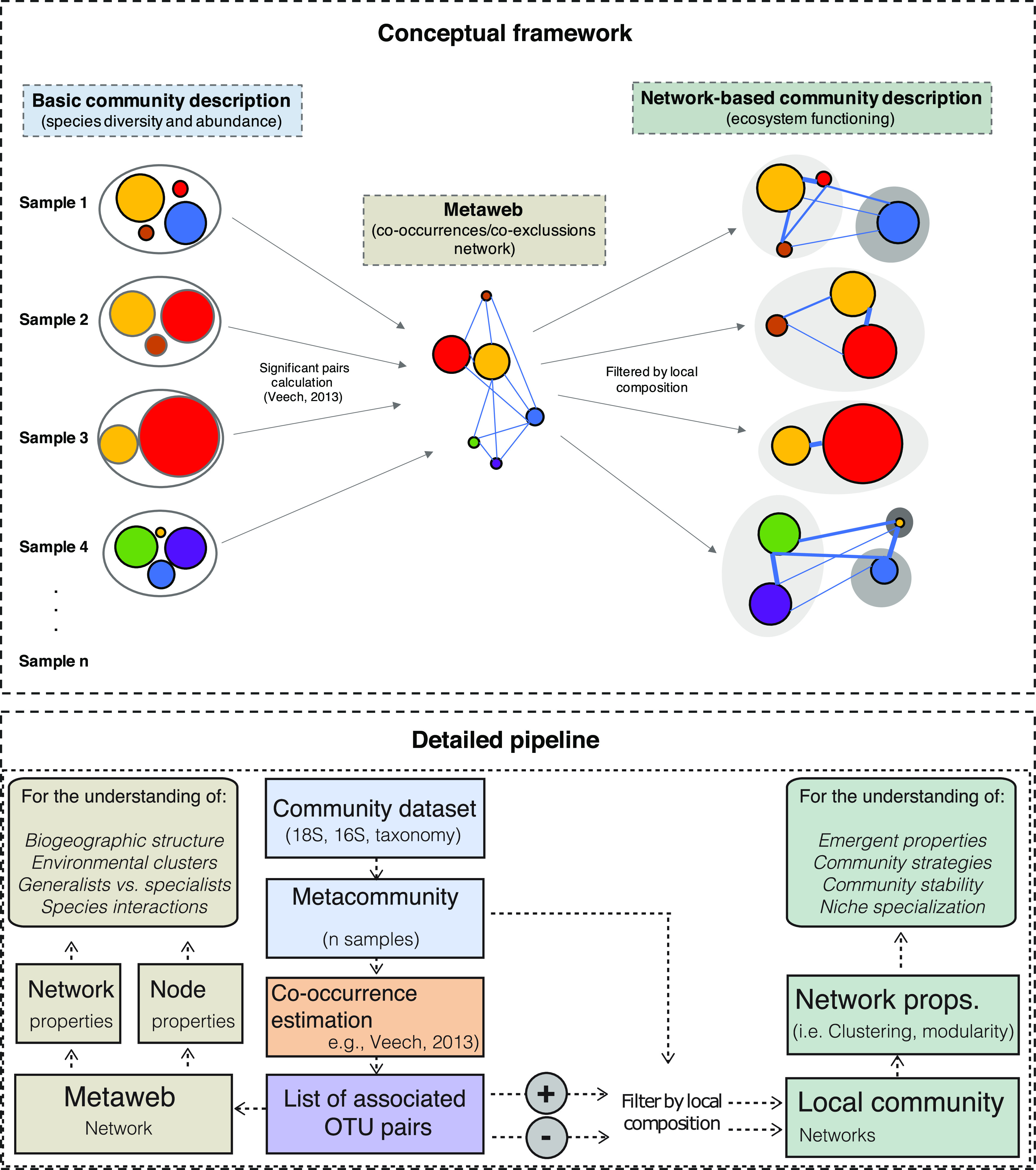
Constructing local community networks from metawebs. Association networks using various spatial scales have been successfully applied to understand the organization and environmental preferences of microbes (15), the fraction of positive trophic interactions (20), generalist versus specialist strategies of particular microbes (81), or even ecological guilds according to potential biotic interactions once the effect of the environment has been removed (Ortiz-Álvarez et al., submitted). This approach is a common strategy in global or regional studies to give general insights of particular metacommunities or larger spatial scales. However, these networks do not inform about the actual arrangements of species in local communities, where species may be loosely or densely connected or display local adaptations to niches or functional guilds. Inferring the local network properties of individual samples characterizes the microbiome of a given sample in terms of its association structure, providing a unique layer of information when studying the biodiversity and stability of a sample or monitoring its evolution in time and during environmental disturbances. Other studies have theorized about how a biogeographical distribution of species interactions is arranged inlocal communities within a given metacommunity or metaweb (82), although this requires the understanding of all the interactions, which is not always possible. Here we combine the metaweb association patterns with local species arrangements to retrieve properties related to particular association arrangements. Considering *n* local communities within a metaweb, we infer significantly associated pairs of species (both positively and negatively associated). These pairs are later sorted for each local arrangement, so only pairs present in each individual sample are considered, to construct local networks, with particular network properties requiring an ecological interpretation. The ecological interpretation varies according to the positive or negative nature of the associations used to construct the networks, leading to quantifying and understanding the effect of ecological disturbance in ecosystems. Now we define the most important network properties used in our study. Connected components are the subnetwork in which any two nodes connect to each other by edges, that lack connection to any other node in the full network. The clustering coefficient is the measure of the degree to which nodes in a graph tend to cluster together in terms of connected triangles (three nodes that are connected with three edges) in the network (73, 74). The average path length is the mean of the minimal number of required edges to connect any two nodes (73, 74). Modularity is the measure of the strength of a partition into modules (groups of nodes). A good network partition harbors a higher proportion of edges inside modules compared to the proportion of edges between them (72).

Using spatial associations based on cooccurrence or coexclusion probabilities is a widespread approach to study why some taxa cooccur or not at different scales. These associations are not direct evidence of ecological interactions (14) but are proxies of the assembly rules at the sampled scale. These assembly rules are often dominated by environmental filters and affinities (15) and dispersal restrictions (16, 17), and also by some biotic interactions (18, 19), although these seem to have a strong bias toward positive facilitation interactions (20). In summary, associations are a combination of these three broad categories, and when they are combined on a metaweb, we obtain a general view of the spatial assembly. These large-scale metawebs have been adequately used to understand taxon affinities to separate ecological niches or geographic clusters or to isolate potential ecological interactions (16; R. Ortiz-Álvarez, V. J. Ontiveros, A. Barberán, J. A. Capitán, D. Alonso, and E. O. Casamayor, submitted for publication). However, these metawebs hardly give an idea of the variety of potential arrangements of local communities, since each local community likely comprises only a fraction of taxa. We argue that integrating the metaweb-inferred associations with the particular subset of taxa from local communities will allow the estimation of network properties to obtain information about the local microbial ecosystem (Fig. 1) serving as community-level properties. Yet, at the same time, it will allow direct comparison among network property communities, even in the absence of common taxa among them. Thus, we aimed at describing the existing relationships between different network properties (e.g., modularity, clustering coefficient) or association distributions (e.g., coexclusion proportions) and how these can provide information of particular assemblies and ecological strategies. For example, studies of the soil rhizosphere show that coexisting functional niches linked to distinct environmental stimuli modulate soil carbon dynamics (21) or that the abundance of antibiotic resistance genes in the topsoil is higher at higher fungal abundances through competitive interactions (22). Also, theoretical studies show that networks can structure in “random” or “small-world” arrangements, and these have particular network properties that can indicate an increased resilience or resistance toward species extinctions (23). Here we will discuss whether network properties could be proxies of such processes or others and suggest further studies to deepen the subject, while indicating whether these could have implications in measuring soil health.

It has been reported that land use and crop management have a strong effect on fungal soil ecosystem functioning (1, 24, 25). When contextualizing community-level properties into management, we will consider that agroecosystems in general, and vineyards in particular, can be managed under three different intensification levels, according to how cultural, biological, and mechanical practices foster the cycling of resources, promote ecological balance, or conserve biodiversity (26): (i) “conventional” management, which allows a wide variety of chemical fertilizers or pesticides; (ii) “organic” management, subjected to strict limits on the use of mineral nitrogen fertilizers and synthetic phytosanitary products; and (iii) “biodynamic” management, as an extreme scenario that rejects the use of mineral nitrogen fertilizers and synthetic pesticides but promotes the use of compost-based fertilization and cover crops, and the use of specific preparations—based on fermented plant materials—to enhance soil fertility and microbial diversity. Since geography, climate, and agriculture management are likely to affect the composition of fungal communities in vineyard soils (27), here we aimed to delve into the functional implications of these changes, understanding whether the structure of local fungal networks can give information about the dominant ecological processes in fungal communities.

To summarize, in this work, we use network properties (i.e., modularity, clustering coefficient) based on large-scale associations (cooccurrences and coexclusions) as proxies of ecological strategies at the community level (e.g., niche differentiation, competition processes). We further evaluate how these properties are affected by environmental conditions and by the agricultural management of vineyard soils in the face of different levels of farming intensification (conventional, organic, or biodynamic management). To achieve this, we first evaluated large-scale structure of metawebs and quantified the influence on community assembly of geography, weather, and network properties. We followed by inferring the network structure of individual samples using the estimated large-scale associations of the two countries studied, United States and Spain. We anticipate that this methodological strategy could be widely applied to understand the effect of environmental disturbances in both natural and human-managed ecosystems and in other fields of interest such as food production or human health.

## RESULTS AND DISCUSSION

### Fungal community assembly in vineyard soils is affected by biogeography and management factors.

Vineyards are human-managed ecosystems, and as one of the most long-lived crops, they are preserved, managed, and exploited for centuries in the same soil. Therefore, vineyard soils can be assumed as stabilized and bounded ecosystems, with its biodiversity molded for decades by the influence of geography, climate, plant-microbe interactions (28–31), but also by human intervention through different types of farming practices which have been demonstrated as a major driver of fungal community composition, impacting vine health and wine fermentation performance (32–34). In this work, we analyzed two data sets of fungal communities, obtained following an internal transcribed spacer (ITS)-amplicon sequencing strategy in vineyard soil samples from the United States and Spain (175 soil samples per country, collected from 2015 to 2018 in vineyards managed with different farming practices: conventional, organic, and biodynamic). The amplicon sequences obtained were then mapped to a list of 31,516 operational taxonomic units (OTUs) with at least 97% identity. Samples had only a fraction of OTU richness, averaging 529 OTUs (minimum [min], 23; maximum [max], 4,999) per soil sample.

**FIG 2:**
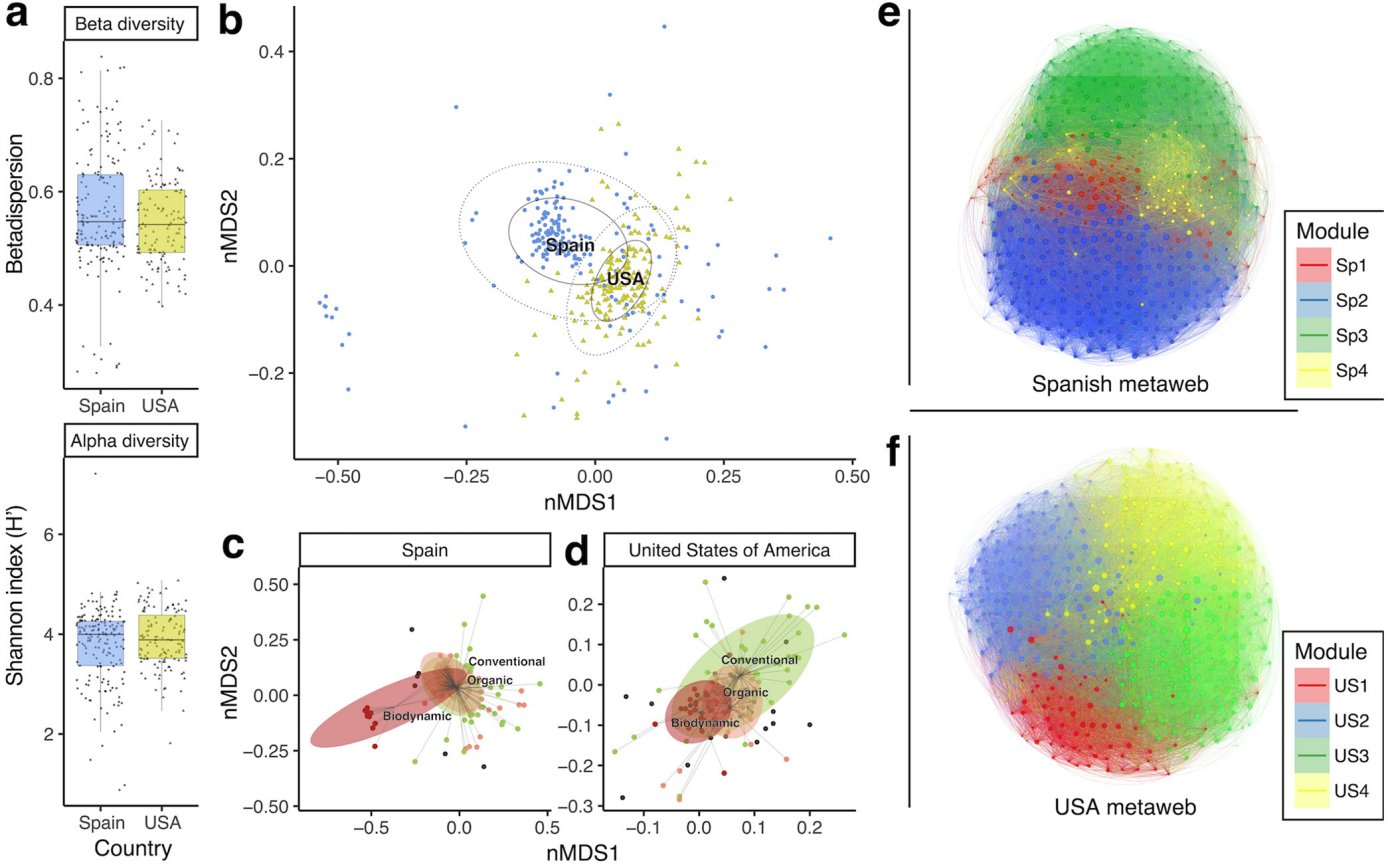
Fungal diversity levels and composition of U.S. and Spain vineyard soil samples. (a) Comparison of alpha diversity (*H*′) and beta diversity (betadispersion) of samples between countries (no significant differences [*P* < 0.05] were found between countries). (b) Nonmetric multivariate ordination (nMDS) of OTU composition, and definition of country-dependent clusters (analysis of similarity [ANOSIM] *R* = 0.31, *P* = 0.001). (c) Multivariate ordination of samples from Spain and definition of management-dependent clusters (ANOSIM *R* = 0.18, *P* = 0.001). (d) Multivariate ordination of samples from the United States and definition of management-dependent clusters (ANOSIM *R* = 0.17, *P* = 0.001). (e) Cooccurrence/coexclusion fungal network of the Spanish metaweb (fraction with the most abundant OTUs) with colored modules (Sp1 to Sp4). (f) Cooccurrence/coexclusion fungal network of the U.S. metaweb (fraction with the most abundant OTUs) with colored modules (US1 to US4). Equivalent numbering of modules in the two metawebs does not imply any compositional equivalence.

We observed that vineyard soils from both countries showed similar alpha and beta diversity ranges (Fig. 2a), and similar proportions of dominant fungal classes (see Fig. S1 in the supplemental material). However, the multivariate ordination of OTU composition showed origin-dependent clusters (Fig. 2b), where the United States and Spain samples were separated by the large geographical distance. In a global study from fungal communities (35), strong biogeographic patterns appear to be driven by dispersal limitation and climate, and our data support this idea. Here we also show that the use of different farming practices for vineyard management has an impact on fungal community composition (Fig. 2c and d), as previously observed by Hartman et al. (36). It is important to highlight that although Spain has the largest organic grape cultivar worldwide (with 100,000 ha of organic grapes) (37), the fungal diversity difference of organic vineyards compared to conventional vineyards is actually undetectable (Fig. 2c). However, in the case of the United States, we observed a more evident effect of this type of management on the soil fungal diversity, showing an intermediate pattern between conventional and biodynamic managed vineyards (Fig. 2d).

10.1128/mSystems.00344-21.1 FIG S1 Taxonomic profile of major classes of fungi separated by country of origin (Spain and the United States [USA]), as classified by UNITE, and summing the relative abundances of the samples from each country. Download FIG S1, PDF file, 0.1 MB. Copyright © 2021 Ortiz-Álvarez et al. 2021 Ortiz-Álvarez et al. https://creativecommons.org/licenses/by/4.0/ This content is distributed under the terms of the Creative Commons Attribution 4.0 International license.

Given these different country-defined sample clusters (Fig. 2b), both data sets were analyzed separately, and two different metawebs were constructed based on two groups of significant cooccurrence/coexclusion probabilities: one for U.S. samples and one for Spain samples (Fig. 2e and f). The study of these metawebs revealed a single connected component with different metaweb properties by continent (modularity, España or Spain [ES] = 0.36, United States [US] = 0.28; clustering coefficient, ES = 0.31, US = 0.57; average path length, ES = 1.91, US = 1.96), a higher observed proportion of cooccurrences in Spain (ES = 0.0696, US = 0.0305) and a similar proportion of coexclusion edges out of the total combinations (ES = 0.0030, US = 0.0028). In addition, both metawebs had four different modules (Fig. 2e and f), each with particular temperature ranges. In metawebs, the majority of associations are derived from the main environmental niches or dispersal (15, 38; Ortiz-Álvarez et al., submitted). In our metawebs, we observed variability in the ranges of maximum temperature for the fungi in each module in both countries, but in a stronger manner in Spain where modules Sp1 and Sp4 (composed by a minority number of OTUs) exhibit opposite temperature preferences (Fig. S2a and b). Since temperature is a critical factor shaping the fungus-mediated soil organic matter decomposition (25, 39) and a major driver of the structure of our fungal communities (Fig. S3), we may expect spatial heterogeneity of this process. The fact that communities have OTUs distributed across a wide range of temperatures is relevant, since drought-resistant fungi control soil organic matter decomposition and its response to temperature fluctuations (25). Therefore, maybe the presence of fungi capable of thriving in broader temperature ranges (such as in the Spanish module Sp4 or U.S. module US2 or US3) is beneficial for soil health. In turn, higher temperatures change carbon allocation within mycorhizal networks (39); hence, that situation may lead to a greater loss of soil carbon and may be detrimental for soil health. A final note regarding modularity of metawebs: these modules show the variability at large spatial scales but are not equally distributed in the local communities. Indeed, each local community has different completeness of each of the modules (Fig. S2c and d) and these vary according to the local modularity value. Hence, the evaluation of local network properties requires an additional layer of discussion.

10.1128/mSystems.00344-21.2 FIG S2 Mean maximum temperature where OTUs thrive classified according to the metaweb modules in Spain (a) and USA (b), and relationship between metaweb module completeness (percentage of OTUs from a module that is present in each local sample) and the local modularity value based on cooccurrences for Spain (c) and USA (d). Download FIG S2, PDF file, 0.2 MB. Copyright © 2021 Ortiz-Álvarez et al. 2021 Ortiz-Álvarez et al. https://creativecommons.org/licenses/by/4.0/ This content is distributed under the terms of the Creative Commons Attribution 4.0 International license.

10.1128/mSystems.00344-21.3 FIG S3 PCA of log-transformed scaled environmental variables separated by country of origin (Spain and USA). Download FIG S3, PDF file, 0.03 MB. Copyright © 2021 Ortiz-Álvarez et al. 2021 Ortiz-Álvarez et al. https://creativecommons.org/licenses/by/4.0/ This content is distributed under the terms of the Creative Commons Attribution 4.0 International license.

### Network properties understood as biomarkers of ecological strategies and environmental filters.

**FIG 3:**
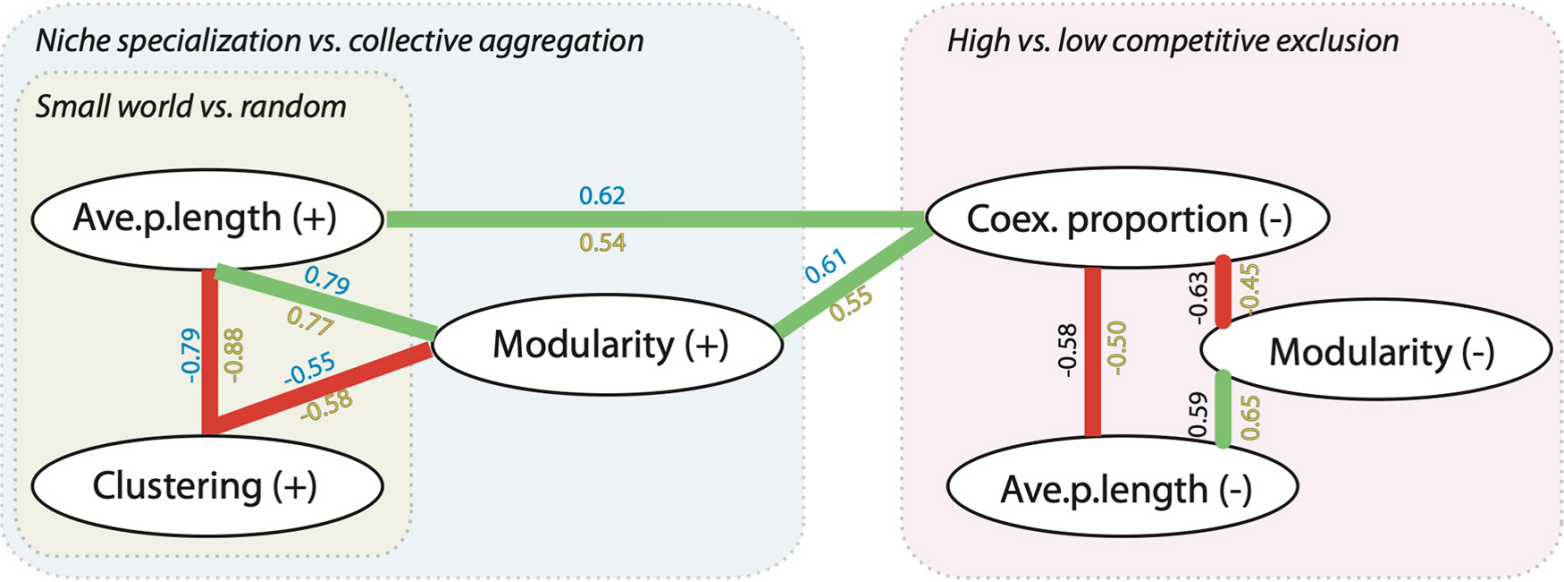
Relationships between network properties. Positive (green) and negative (red) relationships between network properties were obtained based on Spearman’s *r* correlations > |0.5| in at least one country and *P* < 0.01. Cooccurrences (+) and coexclusions (−) are depicted. Relationships within metawebs for Spanish samples have *r* values in blue, and relationships within metawebs for the U.S. samples have *r* values shown in yellow. Interpretation of the properties and their associations are indicated in boxes: small-world versus random networks, niche specialization versus mixed communities, and high versus low competitive exclusion. Values for relationships between properties in a large-scale continental metaweb (merging U.S. and Spain databases) follow similar trends with the following values (Spearman’s *r* correlations > |0.5| and *P* < 0.01): Ave.p.length (+) and Clustering (+) = −0.88; Ave.p.length (+) and Modularity (+) = 0.78; Clustering (+) and Modularity (+) = −0.59; Coexclusion (Coex.) proportion (−) and Ave.p.length (−) = −0.50; Coex. proportion (−) and Modularity (−) = −0.58; Modularity (−) and Ave.p.length (−) = 0.74.

Metawebs constructed using large-scale spatial associations, multivariate techniques, and correlative evidence are often used to interpret overall characteristics of a study system (40). We move forward from this strategy by deriving community-level properties using the large-scale spatial associations and the local community compositions (Fig. 1). By constructing smaller networks for these communities, we are able to classify community types within the whole study area, a common goal when using network theory (41). When evaluating network properties for each community, we observe that the network properties have wider ranges than the overall value of these properties in the whole metawebs, for instance modularity (ES = 0.003 to 0.42, US = 0.08 to 0.31), or clustering coefficient (ES = 0.44 to 0.93, US = 0.34 to 0.66) (see the full list of ranges in Table S1 in the supplemental material). Interestingly, despite the geographic differences, most of the network metrics had similar relationships in the two areas (Fig. 3), indicating a pattern consistency that we find worthy to contextualize. Out of the properties based on cooccurrences, the strongest association was an inverse relationship between clustering coefficient and average path length (Fig. 3), indicating how densely connected are the networks, and thus the degree of association between nodes. This relationship shows a gradient between two alternative situations, which based on network theory can be interpreted as a random network (low clustering, high path length) or a small-world structure (high clustering, low path length) (42). When interpreting these two network properties at the community level, we may gain information about how the actions in one part of the community affect others; for instance, in such theoretical small-world networks, random loss of species is unlikely to affect the overall properties of the network, implying a degree of overall resistance toward potential perturbations (23, 43). In this regard, a densely connected network of cooccurrences may represent a well-integrated collaborative fungi community, where its organisms could have cooperative activities (such as facilitation, syntrophy and/or cross-feeding) (20) and where the loss of one organism may not affect community functionality and hence not be detrimental (43). However, the low clustering situation, which network theory interprets as “random” may not be random but may be simply reflecting a different soil situation as shown by a relationship with another network property: modularity.

10.1128/mSystems.00344-21.8 TABLE S1 Summary of ranges (min, max, mean) for network properties. Download Table S1, XLSX file, 0.01 MB. Copyright © 2021 Ortiz-Álvarez et al. 2021 Ortiz-Álvarez et al. https://creativecommons.org/licenses/by/4.0/ This content is distributed under the terms of the Creative Commons Attribution 4.0 International license.

Modularity indicates the degree of separation of the network into modular components (a fraction of nodes within the network have more edges among each other than with other fractions of nodes in the network). Modularity can successfully separate groups of organisms below the level of community that have a shared mix of associations (Ortiz-Álvarez et al., submitted). This shared mix of associations may be together for multiple reasons. Since associations across the spatial scale mostly reflect the presence of environmental thresholds (20; Ortiz-Álvarez et al., submitted), it makes sense that the primary reason behind modules at large spatial scales are these thresholds (15). However, since we estimate modularity for each single community, with a single set of environmental conditions, there are four possible ways to interpret high modularity. (i) The community has multiple sets of OTUs that may change depending on the environmental situation, for example, by changing weather in different seasons (25). (ii) We may also find sets of OTUs that reflect microscale niches such as topsoil versus soil a few centimeters deeper (21). (iii) Modularity may be indicating independent environmental factors, for example, a group of organisms promoted by a particular pH level, and another group of organisms that is primarily promoted by a particular temperature (15), (iv) Finally, modularity may be an indicator of functional guilds, with each guild with independent affinities (44, 45). With all these possibilities, we can discuss that we are dealing with some kind of “niche specialization,” although we cannot indicate which of the possibilities we are detecting. Interestingly, we observe an inverse relationship between the clustering coefficient (+) with modularity (+) (Fig. 3). Considering the previous interpretation of densely connected networks being those with fungi preferring the same environmental conditions, perhaps those with low modularity are indicative of low niche specialization, indicating fungal communities capable of responding holistically and consistently toward environmental gradients (i.e., temperature [Fig. S2]) or whose members take advantage of metabolic by-products by cross-feeding or facilitation (46). However, their functional capabilities or ranges of reaction may be more limited and less flexible.

In addition to the possibilities discussed above to explain niche specialization, this phenomenon is also one of the main strategies that organisms can pursue to survive over time in highly competitive environments (47), where competition is central in regulating community assembly over time (47, 48). Because of its importance, we aimed to evaluate whether the proportion of coexclusions could be considered an indicator of competition processes. First, we need to consider that we base coexclusions on the premise that a pair of OTUs occur together less than expected at random. In large-scale metawebs, these segregated pairs can indicate different niche specialization (groups of OTUs that are specialized to a particular environment and cannot thrive elsewhere), different geographic locations (a physical dispersal barrier that impedes OTUs from coinciding in a particular sample), or competitive processes (i.e., antifungal and toxin production [49] or differences in resource use efficiency [45]). The estimated coexclusions are a mix of the phenomena mentioned above, and it is not possible to state whether a coexclusion is the consequence of one or more of them. In addition, large-scale associations tend to overemphasize disjoint distributions (14), but in the local networks constructed in our study, there are two particularities. Since each sample belongs to a single locality, it is unlikely that a coexclusion in that community is the consequence of a dispersal difference, and since each sample has a single full set of environmental characteristics, coexclusions in a sample can exist only if (i) the local community has territorial niches assigned (50), (ii) the local community has different OTU groups adapted independently to environmental or functional niches (45), or a combination of the two. Otherwise, since OTUs that should not occur together do occur together, we may interpret them as potential competitors, whose pairs can and have been observed often to coexist (51). Although this strategy does not retrieve the strongest competitors that impede its counterpart to coexist, as shown in controlled simulations (14), it still would retrieve a fraction of them, hence, coexclusion proportion may be indicating some kind of overall competitive equilibrium. Indeed, we observed in our results that highly modular cooccurrence networks (with modularity interpreted as niche specialization) sustain a higher proportion of coexclusions (Fig. 3), which may be quantifying both increased competition processes and stronger niche separations. Furthermore, in the case of fungi, the specialization of functional guilds may be part of the local coexclusions observed. If this were the case, fungi may be competing for the same limiting resource through interference competition (such as in mycorrhizal fungi versus saprotrophs) (45), thus affecting soil carbon dynamics (39, 45).

We argue that we have been able to contextualize three network properties based on cooccurrences/coexclusions to assess part of the ecology of local fungal communities, demonstrating that measuring local network properties substantially increased the proportion of community arrangement variations explained by the other abiotic factors such as temperature (Fig. S4). These results point to the crucial relevance of emergent mechanistic processes on community assembly based on species associations as previously suggested (52). Clustering coefficient, modularity, and the proportion of coexclusions may be critical in the internal ecological mechanisms between the effect of the environment and the influence in ecosystem processes that may ultimately define soil health. Because these three have relationships between each other, we interpret that these are three components that seem to be indicative of the ecological strategies that communities can implement in its assembly. These sides are the ecological stability toward OTU extinctions by having a degree of integration of its members, how the community is separated into environmental niches (hence, affecting stability toward an environmental perturbation), and how likely is that these niches are related to fungal competition processes, which may help to shape these niches.

10.1128/mSystems.00344-21.4 FIG S4 nMDS ordination of soil fungal communities based on Bray-Curtis dissimilarities of OTU composition in Spain (stress = 0.17) (a) and United States (stress = 0.23) (b). The arrows indicate the direction which the meteorological factors, geography and network properties fit the best (using envfit function) onto the nMDS ordination space (only *P* < 0.01 shown). The size of the arrow is proportional to the strength of the correlation of each variable. The right panel (c) shows the percentages of variation in the nMDS ordinations (Spain and USA) explained by the three metadata types through variation partitioning. Variation not explained (residuals) and shared variation between the three metadata types are also shown. Download FIG S4, PDF file, 0.7 MB. Copyright © 2021 Ortiz-Álvarez et al. 2021 Ortiz-Álvarez et al. https://creativecommons.org/licenses/by/4.0/ This content is distributed under the terms of the Creative Commons Attribution 4.0 International license.

### Farming practices are associated with fungal community structure and network properties.

**FIG 4:**
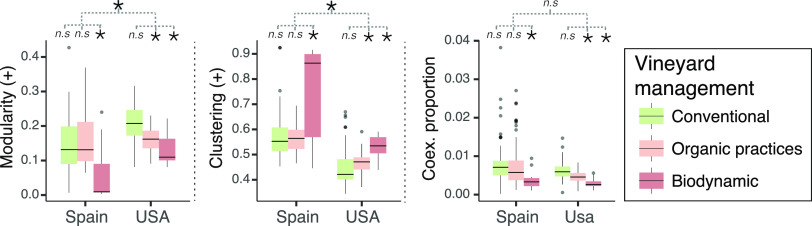
Impact of farming practices in fungal network properties. Boxplot of network properties, (left) Modularity (+), (middle) Clustering (+), (right) Coexclusion proportion (−) under different management practices and countries. For each property, it is indicated if there is a statistically significant difference, according to a two-way ANOVA (n.s., not significant [*, *P* < 0.01]).

The environment affects fungi at the community level, having consequences in community-level properties. Moreover, in vineyards, soil communities are partially shaped by human activities. In fact, Table 1 shows that the farming practices applied in a vineyard are a major predictor of the network properties of soil fungal communities, with estimate values much higher than other relevant environmental factors such as the local temperature, humidity, or wind speed. Our results indicate that management strategies (particularly conventional versus biodynamic approaches) affect network properties of fungal soil communities, following similar trends in the two countries studied. We observed that soils under biodynamic management had higher clustering coefficient (+), lower modularity (+), and lower coexclusion proportion than the conventionally managed soils, with organic managed samples tending to show intermediate values between conventional and biodynamic samples (Fig. 4). In our context, biodynamic-farmed vineyards showed microbial communities closer to (i) small-world networks (higher clustering coefficient [+]) and (ii) mixed (low niche specialization) communities (lower modularity [+]) (Fig. 4, left and middle), properties which are related to enhanced system homeostasis (47) and to higher resistance toward species removal and perturbations. Indeed, land use has been shown to affect the resistance of soil food webs, and in extensively managed grasslands, these webs were more resistant and adaptable to temperature fluctuations and drought (24). Conversely, conventionally managed vineyards were associated with low clustered, highly modular fungal networks (Fig. 4, left and middle) with a larger proportion of coexclusions compared to other management types (Fig. 4, right), as previously observed in root-associated fungal networks from farmlands (53), where increased intensification practices reduced fungal network connectivity. We wonder whether the use of punctual fertilization programs with high doses of specific nutrients, as in conventional farming, may drive a metabolic specialization leading to an arrangement of niches (46), which seems likely given our observations in conventionally managed samples, in contrast to the more densely connected communities under biodynamic management. It should be noted that, for both modularity (+) and coexclusion proportion, the actual scores observed for the samples are, on average, lower than expected by random, according to a null model expectation—independently of the type of management but with a significant trend further away from the results expected by random in biodynamic samples compared with conventional samples (Fig. S5). Opposite values are observed for clustering coefficient (+) scores, where the actual values tend to be higher than expected by random, according to a null model expectation, and with a significant increasing trend—further away from the results expected by random—in biodynamic samples compared with conventional samples. It is noteworthy that, in the case of Spain, only biodynamic samples show clustering coefficient (+) scores different (higher) from those expected by random, highlighting the outstanding structure of fungal communities under this type of management. Ultimately, biodynamic management has been reported to lead to higher-quality grapes in vineyards than conventional management, with organic practices showing an intermediate effect, based on soil fertility, nutrient availability, enzyme activity, and earthworm abundance (26). Taking our results together, we have observed that communities with lower modularity (+) had higher completeness of modules that were associated with wider temperature ranges (Fig. S2). According to the higher clustering coefficient values and lower proportion of coexclusions under biodynamic management (Fig. 4), this management would sustain community resistance toward, at least, temperature fluctuations. A similar result was reported in a grassland experiment, where drought reduced the proportion of negative correlations, and showed that fungi were better adapted to drought than bacteria (54).

**TABLE 1:** Effects of management type, humidity, temperature, and wind speed on modularity, clustering, and coexclusion proportion^*a*^

Fixed effect	Estimated value ± standard error^*b*^		
	Modularity (*r*^2^ = 0.955)	Clustering (*r*^2^ = 0.665)	Coex. proportion (*r*^2^ = 0.999)
Conventional management	−1.637 ± 0.118***	−0.730 ± 0.072***	−5.129 ± 0.335***
Organic management	−1.686 ± 0.115***	−0.736 ± 0.072***	−5.182 ± 0.333***
Biodynamic management	−2.419 ± 0.153***	−0.518 ± 0.074***	−6.056 ± 0.376***
Humidity	−0.126 ± 0.028***	0.048 ± 0.014***	−0.180 ± 0.053***
Temperature	−0.084 ± 0.032**	0.083 ± 0.014***	−0.107 ± 0.054*
Wind speed	−0.013 ± 0.023	−0.012 ± 0.009	0.020 ± 0.035

a In each generalized mixed model, regionality was included as a random effect. Coex. proportion, coexclusion proportion.

b Marginal *r*^2^ are indicated in the table column names. Asterisks indicate a significant contribution of this variable as an estimate on the model: ***, *P* < 0.001; **, *P* < 0.01; *, *P* < 0.05.

10.1128/mSystems.00344-21.5 FIG S5 Impact of farming practices in fungal network properties. Boxplot of network properties, Modularity (+) (a), Clustering (+) (b) , and Coexclusion proportion (−) (c) under different management practices and countries. Estimated properties are compared to a null model. Horizontal lines indicate whether the values are higher (2) or lower (−2) than expected by chance. Between 2 and −2, the estimated properties are not different from the null model. For each property, statistically significant differences according to a two-way ANOVA are indicated (n.s., not significant; *, *P* < 0.01). Download FIG S5, PDF file, 1.9 MB. Copyright © 2021 Ortiz-Álvarez et al. 2021 Ortiz-Álvarez et al. https://creativecommons.org/licenses/by/4.0/ This content is distributed under the terms of the Creative Commons Attribution 4.0 International license.

Because the assessment of fungal biodiversity as soil health indicators cannot be limited only to the determination of diversity indexes (1), we argue that a combination of local network properties is a useful approach to understand soil health. However, diversity indexes may still be useful. In this study, we observe that alpha diversity (*H*′) is higher in the communities with a lower modularity and a lower proportion of coexclusions than sparsely connected communities (*r* = −0.41, *P* < 0.001). This result agrees with the previous interpretations regarding aspects of biodiversity-ecosystem stability (55–58), so communities with the highest diversity may have presumably higher resistance toward perturbation and would tolerate wider temperature variations. In parallel, the coexclusion proportion was associated with lower plant pathogen richness (*r* = −0.28, *P* < 0,001) (Fig. S6a). By using a linear model, we predicted that at the lowest coexclusion proportion values (such as those from biodynamic and organic managements), the probability of the presence of a plant pathogen rises to 80% (Fig. S6b). However, it is essential to note that a higher richness or abundance of pathogens in soils does not always imply a higher risk of disease development. For instance, land management practices, including reduced tillage, may improve soil health by promoting fungal populations with suppressive effects against pathogenic microorganisms (1). Given that organic management and biodynamic management may use tillage systems to compensate for the reduction/absence of chemical fertilizers and phytosanitary products, it is understandable that under organic and biodynamic management, the presence of pathogens may be more likely. To wrap up, environmental conditions, the direct consequences of phytosanitary programs, and the whole fungal community context are the determinants for the quality and health of the soil ecosystem. We suggest that network properties should be taken into account in future in-field studies regarding plant pathogens and natural soil control.

10.1128/mSystems.00344-21.6 FIG S6 Relationship between fungal pathogen richness and coexclusion proportion of the network. (a) Boxplot of coexclusion proportion at different pathogen richness (see Table S2 for a detailed list of the vine pathogens considered). (b) Probability of fungal pathogen presence at different coexclusion proportion values. Download FIG S6, PDF file, 0.7 MB. Copyright © 2021 Ortiz-Álvarez et al. 2021 Ortiz-Álvarez et al. https://creativecommons.org/licenses/by/4.0/ This content is distributed under the terms of the Creative Commons Attribution 4.0 International license.

### Conclusions and perspectives.

**FIG 5:**
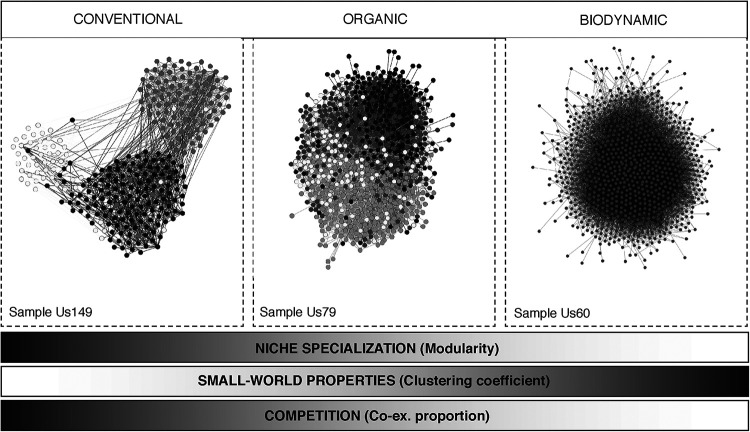
Contextualizing fungal community structure and ecological strategies in vineyard soils based on network properties. Based on our results, the use of contrasting agricultural management systems (conventional versus biodynamic) may lead to different combinations of community-level properties and community structures in vineyard soils. The fungal communities favored under biodynamic management may resemble a community structure close to that in wild cooperation-based environments, as opposed to the specialized environment found in conventionally farmed vineyards. As highlighted in a recent consensus paper (83), the niche specialization found in global soil fungal and bacterial communities and their sensitivity to environmental changes may compromise the future delivery of agroecosystem services. This statement is based on the demonstrated effect that climate change consequences, such as aridity, have on the reduction in the microbial diversity and abundance of soils. This problem may be greater in highly specialized niche-partitioned environments with increased competition/coexclusion, where lower functional redundancy, lower cross-feeding processes, and species loss may have a stronger impact on community stability. We argue that fungal communities closer to small-world and collaborative networks, as in biodynamic managed soils, can be more resistant to the continuously changing environment imposed by climate change and land use than sparser partitioned communities. Thus, community-level properties may be useful to evaluate for soil health in agricultural systems, since soil health reflects the capacity of soil to respond to agricultural intervention while sustaining both the agricultural production and providing other ecosystem services (84). Thus, the biological sustainability of agroecosystems comes from the interaction of the biological processes provided by a diversity of interacting soil organisms and the influence of the abiotic soil environment, with human intervention playing a key role in this interaction.

There is an urgent need for systems-level approaches for understanding agroecosystem functioning and for studying their sustainability in terms of resistance and resilience (1, 59). In this sense, the inference of community-level properties (3) based on association networks seems a successful strategy to follow. Here we have deciphered different ecological strategies that fungal communities adopt in the face of different levels of farming intensification and explored how these may impact soil health in terms of external factors (temperature ranges, management practices) and plant pathogens. On the basis of our findings, we can conclude that even in a single ecosystem, human intervention can determine two alternative fungal community assembly strategies: a collaborative well-mixed habitat in soils under biodynamic management with potentially higher resistance toward, at least, temperature variations, or a more divided habitat, with fungi belonging to more niches but with lower reaction range to temperature in soils under conventional managements. We interpret these situations as alternative equilibrium states of communities (Fig. 5), with those from biodynamic managements closer to small-world networks, acknowledging the critical impact of microbial associations on microbial community assembly, moving away from the highly specialized niche-partitioned environments that characterize soils under conventional management. Scaling up to the broader picture, several authors have proposed that two of the major forces driving the current global change in ecosystems functioning—habitat modification and climate change—are expected to select habitat generalists instead of those habitat specialists with lower biodiversity levels and a higher niche partitioning (60, 61). Under this framework (Fig. 5), our results may guide future in-field studies on the biodiversity-stability hypothesis, its relevance for agriculture sustainability, and how human intervention may drive a better future for agroecosystems. Here we want to recognize some limitations of our survey, since unfortunately, we did not evaluate interesting physical-chemical aspects of the soils analyzed (e.g., pH, nutrient availability, etc.) that would help us in understanding the actual reasons explaining the significant patterns observed across management types. Thus, we encourage further experimental studies in agriculture soils under different management types under controlled conditions, including longitudinal (time series) studies in conversion scenarios (changing from conventional to organic or biodynamic farming) to unravel the main biotic and abiotic factors driving the fungal community assembly patterns in terms of their local network properties. For instance, evaluating how network properties change during time series may give clear indications about the resistance and resilience of fungal communities or shed light into the dynamics of soils under different anthropogenic disturbances. For now, the contextualized network properties based on large spatial-scale associations may be used as biomarkers to measure the effect of farming practices or temperature change consequences in the health status of soils. Given the key role that microorganisms play in agri-food systems in general, and in the wine industry in particular, these findings are useful for establishing monitoring programs of crop-associated microbial diversity, supporting the work of alliances such as the Soil Health Institute (soilhealthinstitute.org), the USDA (www.nrcs.usda.gov/wps/portal/nrcs/main/soils/health/), or the Global Initiative of Crop Microbiome and Sustainable Agriculture (www.globalsustainableagriculture.org), while promoting soil healthiness through agriculture sustainable strategies. We hope that this work may inspire other researchers on the use of network properties at the community level in microbiomes at several different contexts, from the human microbiome (to understand the role of microbiome in health and disease) to the microbial communities found in extremely anthropogenic ecosystems such as food fermentations or industrial bioreactors.

## MATERIALS AND METHODS

### Sample collection, environmental, and management metadata.

This study is a microbial amplicon-based survey that includes a total of 350 soil samples from vineyards from the United States (175 samples; mostly California and southern states) and Spain (175 samples) collected in the period from 2015 to 2018 (see Fig. S7 in the supplemental material). All the samples were of topsoil, taken at a 30-cm distance from the vine trunk, within a depth between 5 and 10 cm. Each sample from a single block was made pooling together topsoil from three random spots in each block. For these samples, we collected geographic location (latitude, longitude, and altitude), and extracted meteorological metadata from the Dark Sky API site (https://darksky.net/poweredby/): climatic information (precipitation intensity, precipitation probability, maximum temperature, minimum temperature, dew point, humidity, environmental pressure, wind speed, wind bearing, wind gust, cloud cover, and UV index). Regarding management systems, vineyards considered “organic” are subject to the regulation of the USDA in the United States (62) and European Commission (63) in Spain. In brief, organic methods integrate cultural, biological, and mechanical practices that foster cycling of resources, promote ecological balance, and conserve biodiversity. Synthetic fertilizers, sewage sludge, irradiation, and genetic engineering may not be used. Vineyards considered “biodynamic” follow the certification described by the Demeter association (https://www.demeter-usa.org). Biodynamic farms aspire to generate their own fertility through composting, integrating animals, cover cropping, and crop rotation. Finally, vineyards considered “conventional” follow a farming system, using a variety of synthetic chemical fertilizers, pesticides, herbicides, and other continual inputs. We asked landowners to provide information about the crop management system (conventional, organic, or biodynamic practices) and compared them when available (124 samples from the United States [conventional {*n* = 65}, organic {*n* = 39}, and biodynamic {*n* = 20}] and 172 samples from Spain [conventional {*n* = 78}, organic {*n* = 79}, and biodynamic {*n* = 15}]).

10.1128/mSystems.00344-21.7 FIG S7 Map of sampling locations. Geographical distribution of sampling locations in USA (a) and Spain (b) and by management practices: conventional (green), organic (orange), and biodynamic (red). Maps were prepared using the software QGIS with the Google base map. Download FIG S7, PDF file, 13.9 MB. Copyright © 2021 Ortiz-Álvarez et al. 2021 Ortiz-Álvarez et al. https://creativecommons.org/licenses/by/4.0/ This content is distributed under the terms of the Creative Commons Attribution 4.0 International license.

### DNA extraction protocol and sequence filtering.

Soil samples were stored at −80°C until DNA extraction. DNA extraction was performed using the DNeasy PowerLyzer PowerSoil kit (Qiagen). A complete overview of all the samples used in this study and their origin is reported in Table S3 in the supplemental material and in BioProject PRJNA672044 metadata (see Fig. S7 for an overview of the geographical distribution of samples. Maps were prepared using the software QGIS with the Google base map). Libraries were prepared following the two-step PCR protocol from Illumina and sequenced on an Illumina MiSeq using paired-end sequencing (2 × 300 bp). Libraries were prepared by the ITS1 region using Biome Makers custom primers (64). Raw files are available under BioProject accession number PRJNA672044. Raw sequences were analyzed using Vsearch using default parameters (65). Briefly, raw paired-end fastq sequences were merged, filtered by an expected error of 0.25, dereplicated, and sorted by size. We filtered out chimera sequences and clustered nonsingleton sequences into 97% identity OTUs. All combined sequences were then mapped to a list of 31,516 OTUs with at least 97% identity, resulting in an OTU table with 54,738,544 sequences, averaging 156,395 sequences per soil sample (min, 21,232; max, 1,213,767). Samples had only a fraction of OTU richness, averaging 529 OTUs (min, 23; max, 4,999) per soil sample. OTUs were classified with the UNITE database with the UTAX pipeline (66).

10.1128/mSystems.00344-21.10 TABLE S3 Sample and metadata list (country and management, more in BioProject accession number PRJNA672044) Table S3, XLSX file, 0.05 MB. Copyright © 2021 Ortiz-Álvarez et al. 2021 Ortiz-Álvarez et al. https://creativecommons.org/licenses/by/4.0/ This content is distributed under the terms of the Creative Commons Attribution 4.0 International license.

### Network analyses for the estimation of community-level properties.

For the estimation of network properties first, we prepared the microbial community data set. Before starting this part of the pipeline, we rarefied samples to a sequencing depth of 20,000 sequences; so all OTUs had equal detectability in all the samples. Then, we filtered out the OTUs with the lowest occurrence, so we kept only those OTUs that appeared in at least 2% of the samples, keeping 5,753 OTUs in Spain and 4,784 OTUs in the United States. We checked for a potential effect of not using all the species of the metaweb using a Mantel test of Bray-Curtis dissimilarities, showing that the filtered communities represented the full local communities adequately. Then, because of current debates on the appropriate use of covariance/correlation methods to infer cooccurrences from microbial community data (67), we chose to transform data to presence/absence, and apply a conservative strategy: the probabilistic method of Veech (68). In short, the Veech model estimates the probability of two species cooccurring or coexcluding each other, at a frequency less or greater than the observed frequency if the two species were distributed independently among sites. In this regard, it is critical to avoid varying sequencing depths and detectability bias (69), which we bypass by rarefying our data set. Out of all the existent methods to estimate cooccurrences, this method is among the most conservative, does not rely on correlations (70), is fast and analytically exact, and does not assume a prior network structure.

Finally, we retrieved significant pairwise cooccurrence and coexclusion probabilities (*P* < 0.05) separately for samples from the United States and Spain. The full list of positive and negative significantly associated pairs represents the potential for interactions in the complete metaweb and/or environmental distributions. The two lists of positive and negative pairs were transformed into two species matrices representing the possibility of cooccurrence/coexclusion in the whole metaweb. To estimate network properties in each local sample, the two metaweb-based species matrices were subsequently subset into 350 matrices containing only the species occurring in each of the individual samples. It should be noted that this filtering step can be done with pairwise lists of cooccurrences estimated in different manners, such as with covariance/correlation cooccurrence estimation methods such as SparCC or SPIECeasi (67). Each of these matrices were transformed into undirected networks using the R package igraph (71), where nodes represent species and edges represent statistically significant cooccurrences/coexclusions. For each network, we estimated the following properties as implemented in the r package *igraph* (71): the number of connected components, modularity using the cluster walktrap algorithm (72), clustering coefficient defined as average transitivity (71, 73), and average path length (74) (a larger-scale metaweb, considering both U.S. and Spain samples, was punctually used to calculate the relationship between network properties in a unique large-scale continental context; results reported in the legend of Fig. 3). We also calculated the proportion of cooccurrences and coexclusions observed out of the total number of combinations of all the OTUs in the sample. A full representation of the process followed is displayed in Fig. 1 (part of this methodology is in a pending patent [75]). All networks were drawn with *gephi* (76).

### Statistical analyses.

For the estimation of alpha diversity and for the cooccurrence estimation, we rarefied samples to 20,000 sequences per sample. We assessed whether weather or management had an effect on network properties through Spearman correlations and Kruskal-Wallis tests, respectively. To estimate the relative contribution of weather, geographic location, and network properties in explaining the heterogeneity in the fungal metawebs, we performed a variation partitioning analysis using the nonmetric multidimensional scaling (nMDS) two-dimension scores as the response variables. The three sets of variables were subject to a forward selection procedure, removing colinear variables, prior to their use as explanatory groups of variables (77). To evaluate how environmental properties (data normalized as the mean deviance per country) and farming practices (both defined as fixed effects) may predict local network properties, we performed a generalized linear mixed model (GLMM) with regionality (USA_northwest, USA_southwest, USA_east, Spain_west, Spain_east, and Spain_islands) as a random effect. In addition, we confirmed the effect of farming practices in local network properties by performing a two-way analysis of variance (ANOVA) (including the country as an independent variable). To evaluate the degree in which local network properties deviate from a null model expectation, matrices containing only the species occurring in each of the individual samples were randomized across the metaweb cooccurrence/coexclusion matrices. We then calculated the number of standard deviations that the observed property is from the mean of the null distribution (1,000 randomizations). We further studied whether we could predict the presence or absence of plant pathogens (using a curated list of vineyard pathogens; see Table S2), by quantifying the total number of plant pathogens. We further used a model to calculate predicted probabilities of presence of pathogens, by fitting variables [Transitivity (+), Modularity (+), Ave.p.length (−) and coexclusion proportion] into a generalized linear model (GLM) using a binomial distribution. Statistics were calculated in the R environment using packages *base*, *vegan* (78), and *GUniFrac* (79) and drawn in *ggplot2* (80).

10.1128/mSystems.00344-21.9 TABLE S2 Disease and pathogen list considered. The presence of that particular pathogen in our data set is included. Download Table S2, PDF file, 0.04 MB. Copyright © 2021 Ortiz-Álvarez et al. 2021 Ortiz-Álvarez et al. https://creativecommons.org/licenses/by/4.0/ This content is distributed under the terms of the Creative Commons Attribution 4.0 International license.

### Data availability.

Raw files are available under BioProject accession number PRJNA672044.
