# Supplementary figures and images for: Network Properties of Local Fungal Communities Reveal the Anthropogenic Disturbance Consequences of Farming Practices in Vineyard Soils
Source: mSystems. 2021 May 4;6(3):e00344-21. doi: 10.1128/mSystems.00344-21 (PMC8269225; doi:10.1128/mSystems.00344-21)

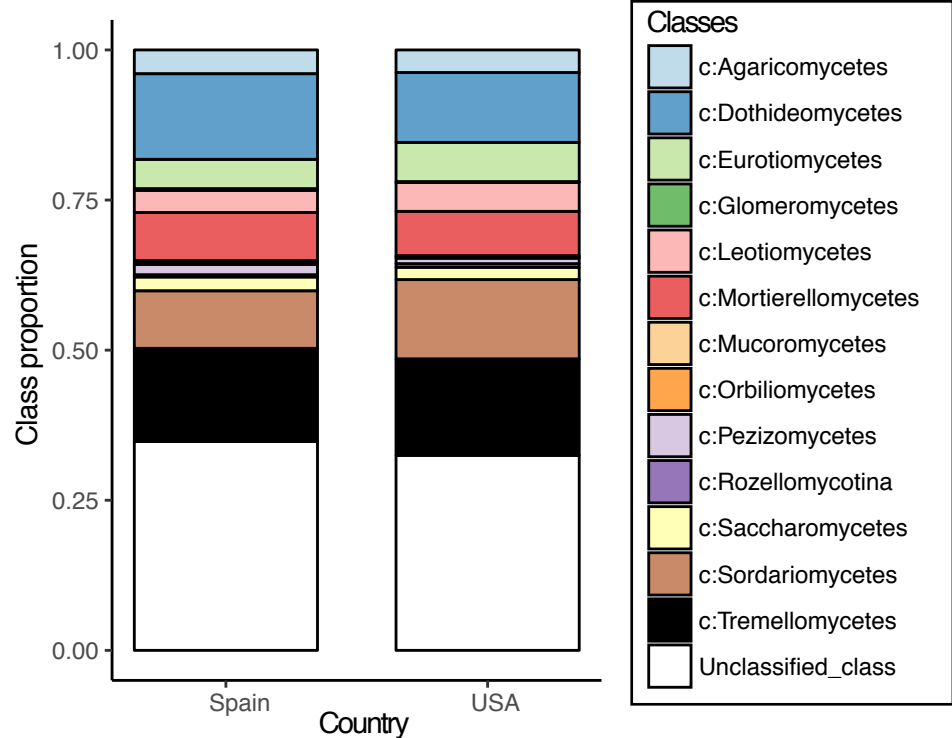

Supplement: FIG S1 [file msystems.00344-21-sf001.pdf]

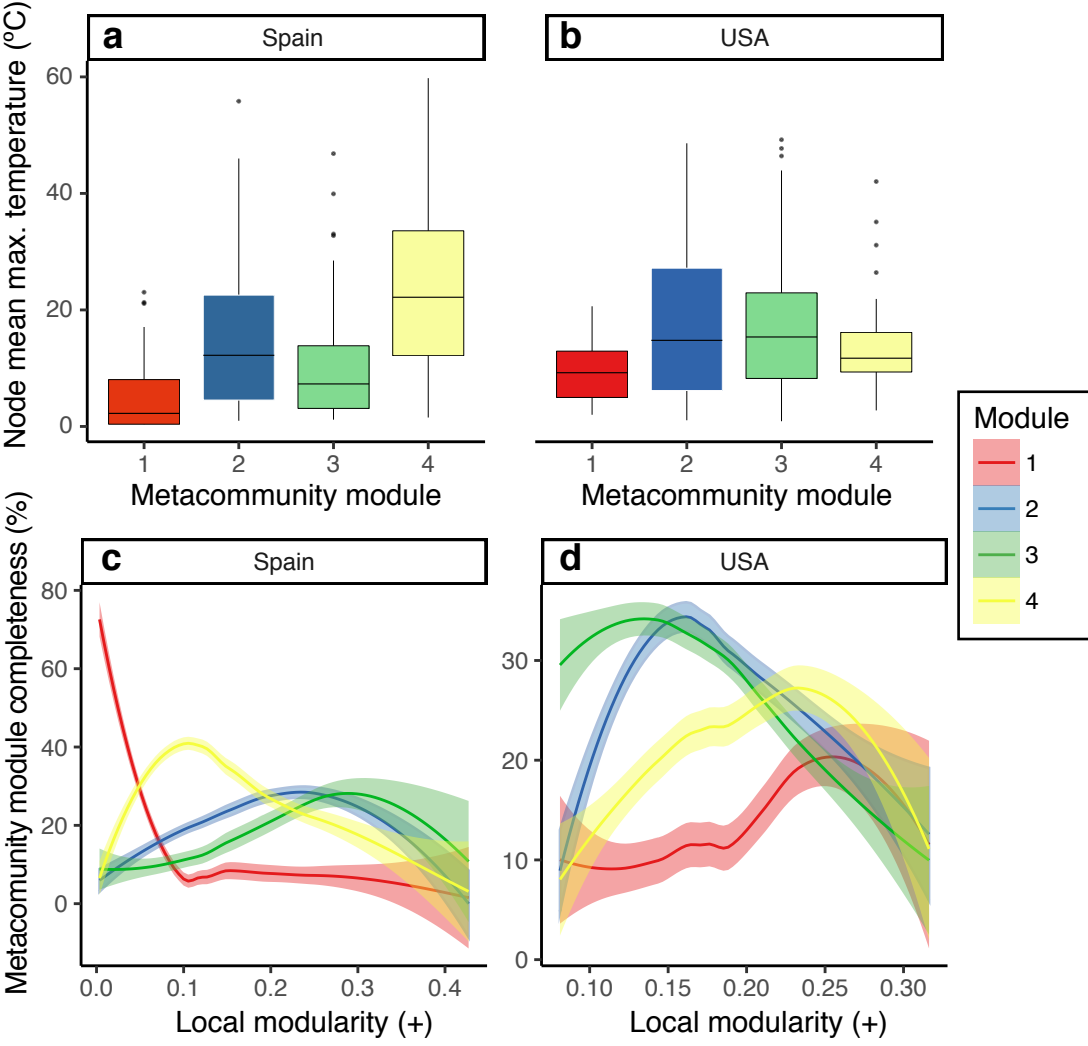

Supplement: FIG S2 [file msystems.00344-21-sf002.pdf]

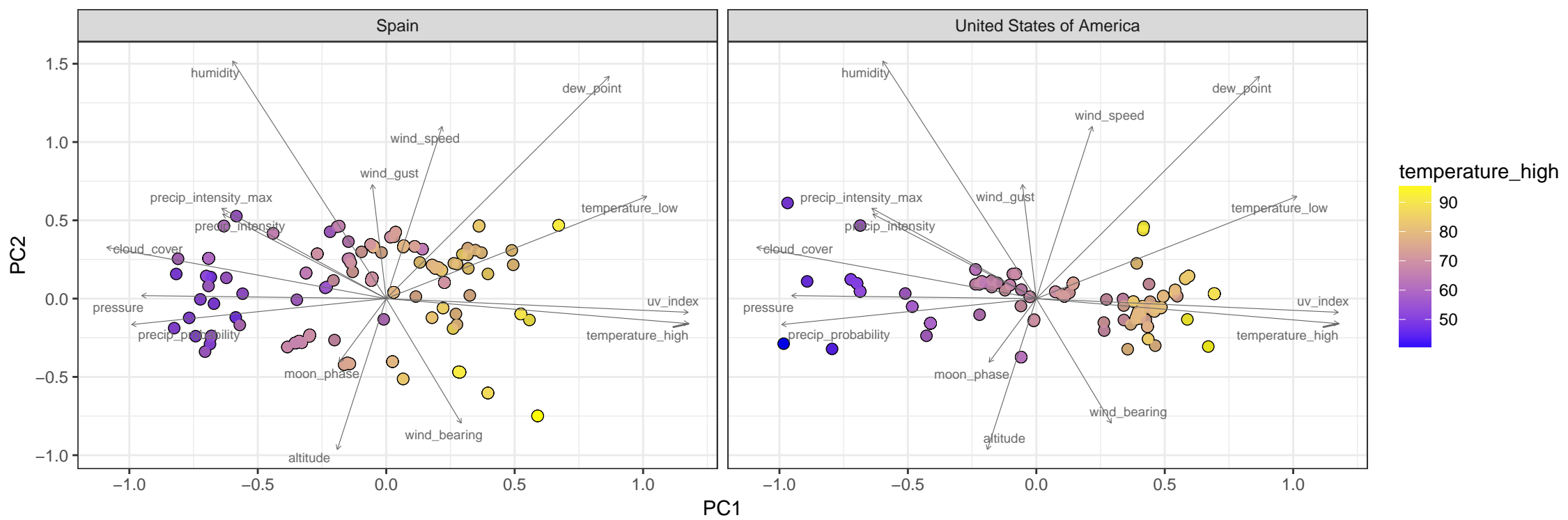

Supplement: FIG S3 [file msystems.00344-21-sf003.pdf]

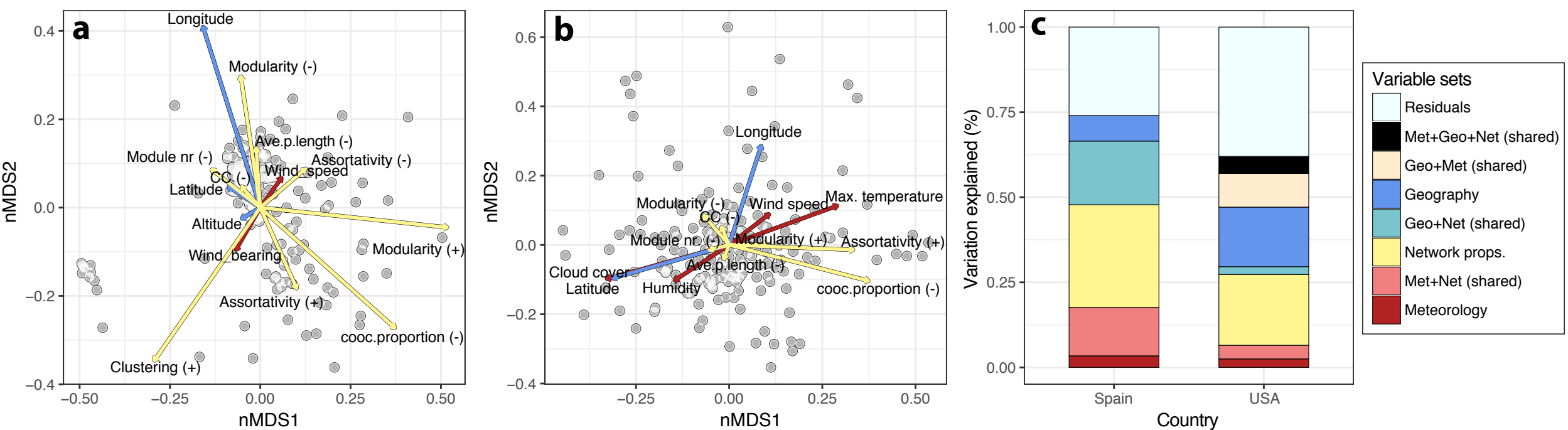

Supplement: FIG S4 [file msystems.00344-21-sf004.pdf]

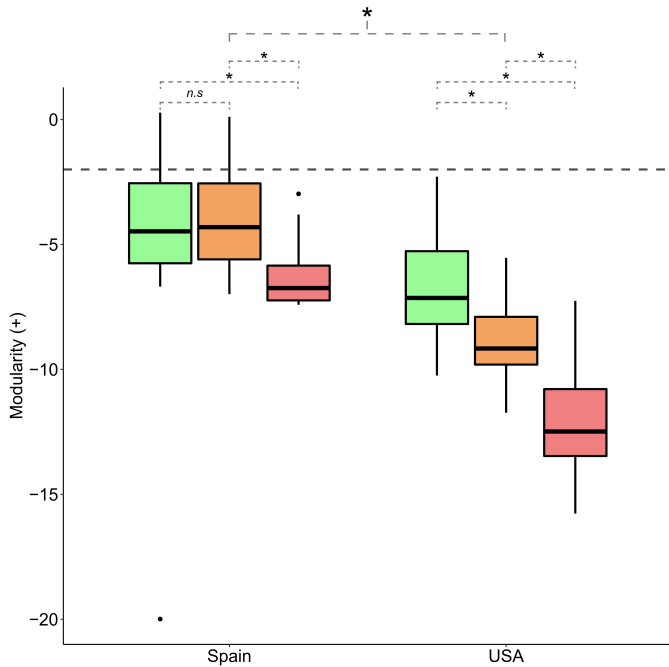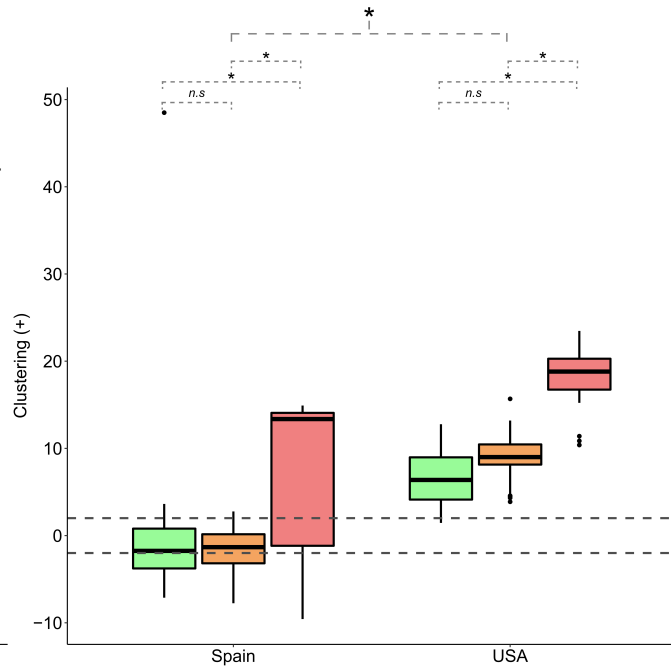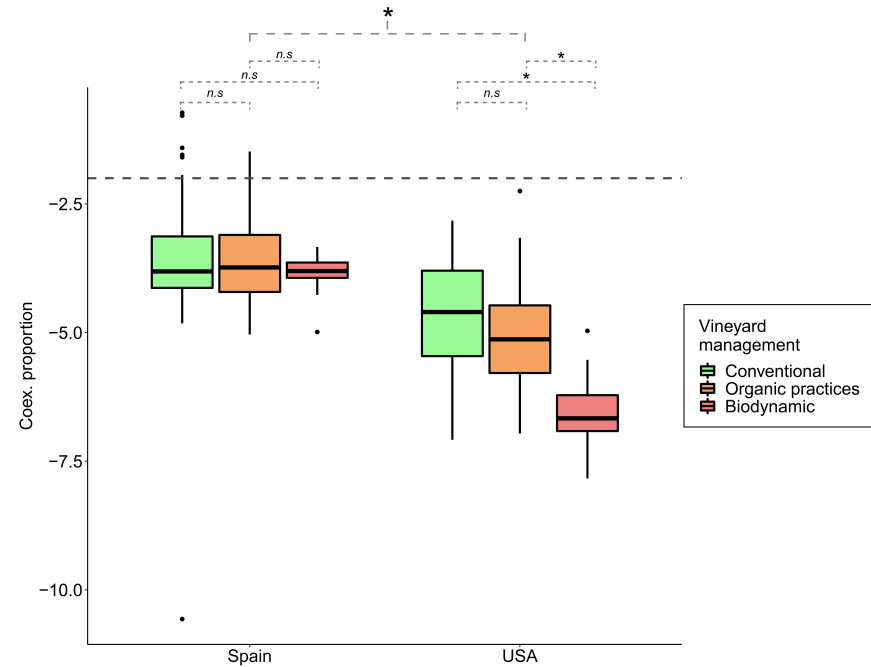

Supplement: FIG S5 [file msystems.00344-21-sf005.pdf]

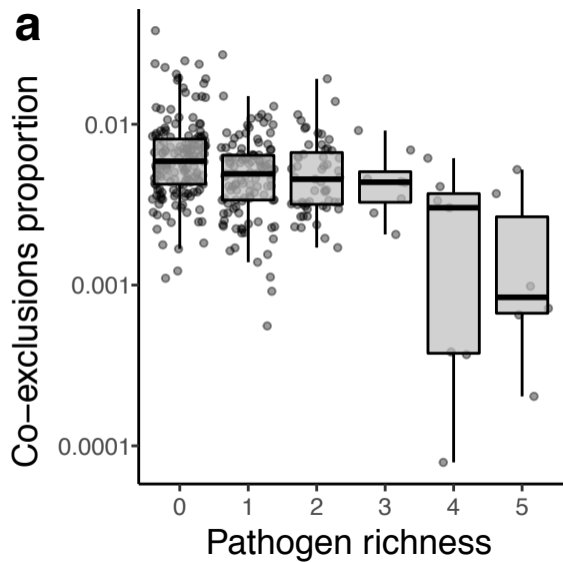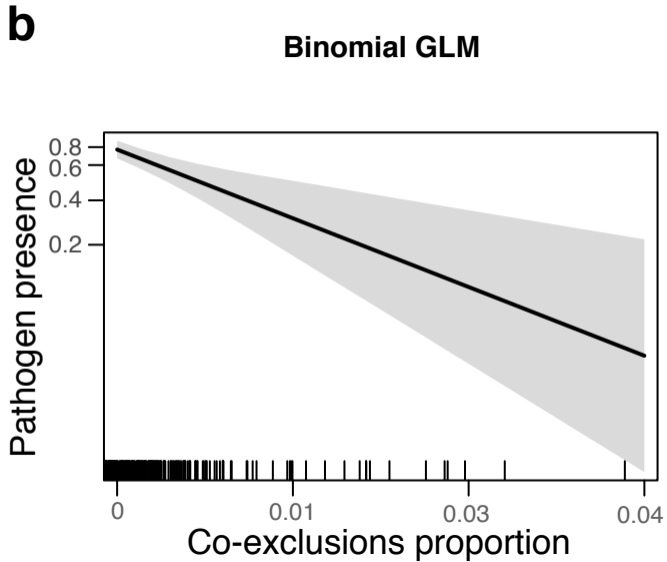

Supplement: FIG S6 [file msystems.00344-21-sf006.pdf]

**A**

115°W

42°N

USA northwest

USA southwest

USA east

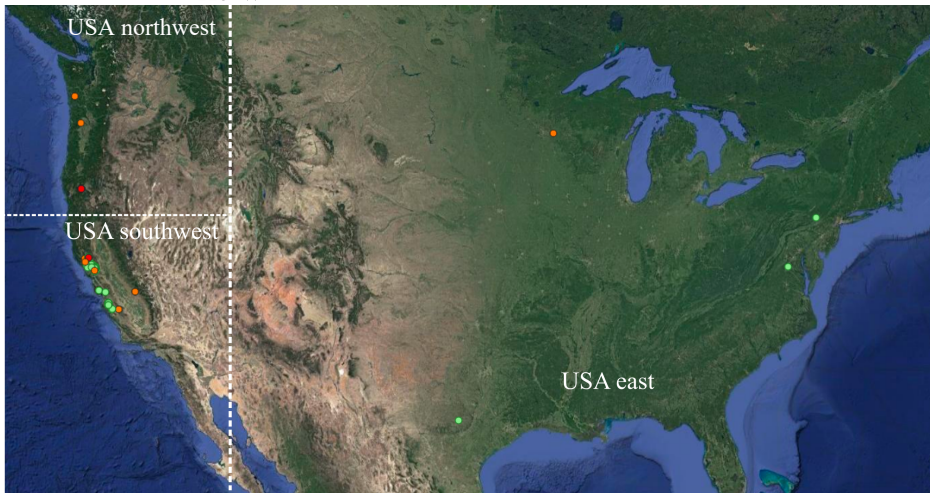**B**

3.8°W

Spain east

Spain west

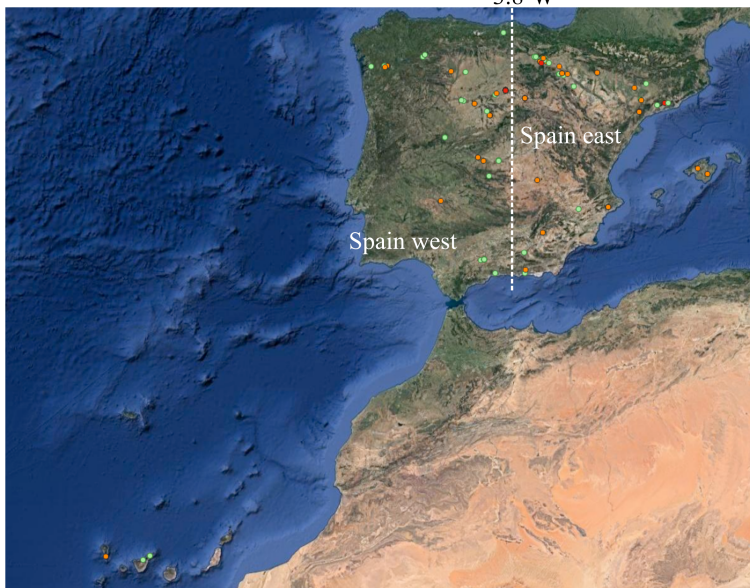

Supplement: FIG S7 [file msystems.00344-21-sf007.pdf]
