# Supplementary material for: Network Properties of Local Fungal Communities Reveal the Anthropogenic Disturbance Consequences of Farming Practices in Vineyard Soils
Source: mSystems. 2021 May 4;6(3):e00344-21. doi: 10.1128/mSystems.00344-21 (PMC8269225; doi:10.1128/mSystems.00344-21)
Supplement: TABLE S2 [file msystems.00344-21-st002.pdf]

**Disease**	**Pathogen sp.**	**presence**
Armillaria root rot	*Armillaria mellea*	present
Aspergillus rot	*Aspergillus carbonarius*	present
Black foot disease	*Ilyonectria robusta*	present
Black foot disease	*Campylocarpon fasciculare*	present
Black foot disease	*Ilyonectria liriodendri*	present
Black foot disease	*Dactylonectria estremocensis*	present
Black foot disease	*Campylocarpon pseudofasciculare*	not present
Botryosphaeria dieback	*Botryosphaeria dothidea*	present
Botryosphaeria dieback	*Lasiodiplodia missouriana*	present
Botryosphaeria dieback	*Neofusicoccum parvum*	present
Botryosphaeria dieback	*Neofusicoccum australe*	not present
Botrytis bunch rot	*Botrytis cinerea*	not present
Esca Complex	*Phaeomoniella chlamydospora*	present
Esca Complex	*Phaeoacremonium minimum*	present
Esca Complex	*Phaeoacremonium hispanicum*	present
Esca Complex	*Fomitiporia aethiopica*	not present
Esca Complex	*Phaeoacremonium inflatipes*	not present
Esca Complex	*Stereum hirsutum*	not present
Eutypa dieback	*Eutypella citricola*	present
Eutypa dieback	*Cryptovalsa ampelina*	present
Eutypa dieback	*Diatrype stigma*	present
Eutypa dieback	*Eutypa lata*	not present
Petri disease	*Cadophora luteo-olivacea*	present
Phomopsis dieback	*Diaporthe ampelina*	present
Verticillium wilt	*Verticillium dahliae*	present
